# Soluble PD-1 aggravates progression of collagen-induced arthritis through Th1 and Th17 pathways

**DOI:** 10.1186/s13075-015-0859-z

**Published:** 2015-11-25

**Authors:** Cuiping Liu, Juean Jiang, Li Gao, Xiaoting Wang, Xiaohan Hu, Min Wu, Jian Wu, Ting Xu, Qin Shi, Xueguang Zhang

**Affiliations:** Jiangsu Institute of Clinical Immunology, First Affiliated Hospital of Soochow University, Suzhou, 215006 China; Jiangsu Province Key Laboratory of Stem Cell Research, Soochow University, Suzhou, 215006 China; Department of Rheumatology, Third Affiliated Hospital of Soochow University, Changzhou, 213003 China

## Abstract

**Introduction:**

The programmed cell death 1 (PD-1) protein is a critical regulator of T-cell activation and is also an important therapeutic target for autoimmune diseases. Little is known about the regulation and functional properties of the soluble PD-1 (sPD-1) variant. The aim of this study was to examine the role of sPD-1 in the regulation of human and murine rheumatoid arthritis (RA).

**Methods:**

Expression of cytokines and sPD-1 in sera, synovial fluid, and peripheral blood (PB) mononuclear cells of patients with RA were analyzed by enzyme-linked immunosorbent assay and quantitative polymerase chain reaction. PD-1 function was assessed in PB T cells after stimulation of the cells with anti-CD3 and PD-L1-Fc to crosslink PD-1. Recombinant PD-1-Fc was injected intraperitoneally into DBA/1 mice with collagen-induced arthritis (CIA) to analyze the function of sPD-1 in vivo.

**Results:**

High concentrations of sPD-1 were found in sera and synovial fluid of patients with RA. The levels of serum sPD-1 were significantly correlated with titers of rheumatoid factor (RF) (*r* = 0.306, *p* = 0.005) and 28-joint Disease Activity Score (*r* = 0.545, *p* < 0.001). Further characterization of sPD-1 revealed that it functionally blocked the inhibitory effect of membrane-bound PD-1 on T-cell activation. Interferon γ, tumor necrosis factor α, and interleukin 17A were identified as inducers of sPD-1 in vitro. Moreover, PD-1-Fc enhanced proinflammatory cytokine expression, generation of Th1 cells and Th17 cells, and joint pathology in a CIA model.

**Conclusions:**

sPD-1 regulates peripheral T-cell responses in both human and murine RA. Thus, sPD-1 may represent an additional biomarker or target in immunomodulatory therapy for RA.

## Introduction

Rheumatoid arthritis (RA) is one of the most common autoimmune diseases, affecting 1 % of the population worldwide [1, 2]. Pathogenic T cells, such as Th1 and Th17 cells, are considered to be critical to the initiation and maintenance of the disease [3–5]. These cells are thought to be triggered locally in an antigen-specific manner, resulting in breakdown of tolerance, synovial inflammation, and autoantibody production [6–9].

Programmed cell death 1 (PD-1; also called CD279), which is inducibly expressed on CD4^+^ T cells, CD8^+^ T cells, natural killer T cells, B cells, and activated monocytes, is a type I membrane protein that delivers inhibitory signals to T cells upon binding of its ligands PD-L1 or PD-L2 [10–13]. PD-1 has been shown to be important for self-tolerance, because spontaneous autoimmune diseases develop in *PD-1*^−/−^ mice [14–16]. In addition, genetic studies revealed that there is an association between polymorphisms in the *PDCD1* gene (which encodes PD-1) and susceptibility to autoimmune diseases [17–19], suggesting that PD-1 may play an important role in the development of autoimmune diseases. PD-L1 is widely expressed in activated endothelial and epithelial cells, and it is therefore thought to be important for the fine-tuning of lymphocyte activation at the level of synovial tissue [20, 21]. Increased numbers of PD-1^+^ and PD-L1^+^ cells were found in the synovium of patients with active RA [22–24].

There are four alternatively spliced *PDCD-1* messenger RNA (mRNA) transcripts in addition to the full-length isoform (flPD-1): PD-1 lacking exon 2 (PD-1Δex2), PD-1 lacking exon 3 (PD-1Δex3), PD-1 lacking exons 2 and 3 (PD-1Δex2,3), and PD-1 lacking exons 2, 3, and 4 (PD-1Δex2,3,4). Soluble PD-1 (sPD-1) is encoded by PD-1Δex3, which retains the extracellular domain but lacks the transmembrane domain [25]. Previous studies have shown that sPD-1 promotes T-cell responses by blocking the PD-1/PD ligand pathway [26–31]. Although the function of sPD-1 in antitumor and antiviral immunity has been studied extensively [26–30], its clinical relevance and function in RA is unknown. It was reported that sPD-1 occurred at high concentrations in sera and synovial fluid (SF) of patients with RA, and PD-1 levels were found to correlate with titers of rheumatoid factor in (RF) patients with RA [32, 33].

We designed the present study to determine the role of sPD-1 in RA and to test the hypothesis that overexpression of this molecule may contribute to T-cell hyperactivity within the inflamed joint. We examined the clinical significance of sPD-1 in patients with RA by determining sPD-1 levels in serum samples. Recombinant fusion proteins corresponding to the extracellular domains (inclusive of the PD-1Δex3 variant) of PD-1 molecule were tested in T-cell proliferation assays using RA-derived peripheral blood mononuclear cells (PBMCs). The role of sPD-1 in RA was further studied by generating collagen-induced arthritis (CIA) in DBA/1 mice and by using PD-1-Fc to block PD-1 signaling in vivo. Our data suggest that sPD-1 may be a promising biomarker for diagnosing and determining the prognosis of RA. sPD-1 and inflammatory mediators of patients with RA significantly attenuated or reversed T-cell suppression mediated by PD-L1-Fc, verifying that sPD-1 acts as a natural blocker of PD-1/PD-L1 signaling and that soluble factors may interfere with this negative pathway.

## Materials and methods

### Patients and specimens

A total of 83 patients with RA were included in the study (Table 1). All patients fulfilled the American College of Rheumatology criteria for RA. This group included 61 females and 22 males with mean disease duration of 12.1 ± 8.0 years. The mean age of the patients was 58.30 ± 13.01 years. They were recruited from inpatient and outpatient clinics at the rheumatology departments of the First and Third Affiliated Hospitals of Soochow University. Disease history was recorded for all patients, including presenting symptoms, affected joint counts, and medication history. The activity of disease was evaluated by calculation of 28-joint Disease Activity Score (DAS28) [34]. The level of RA disease activity can be interpreted as low (Lo-RA; 2.6 ≤ DAS28 ≤ 3.2), moderate (Mo-RA; 3.2 < DAS28 ≤ 5.1), or high (Hi-RA; DAS28 > 5.1), and a DAS28 < 2.6 can be considered as remission (Re-RA), according to the European League against Rheumatism criteria. According to extraarticular involvement, the subjects were divided into patients with RA with limited joint manifestations and those with extraarticular manifestations. Eight of the patients received methotrexate (MTX) therapy (10 mg/week for 20 weeks by oral administration, including follow-up periods of 16 and 32 weeks). None of the patients had received steroid or immunosuppressive drugs within 1 year before the study period. Complete sets of paired SF and peripheral blood were obtained from 15 of the 83 patients for paired analyses. Additional sets of SF and paired serum specimens (no cells) derived from the remaining 68 patients with RA were used only for analyses of protein concentrations of sPD-1 by enzyme-linked immunosorbent assay (ELISA). Complete sets of paired SF and peripheral blood samples from a total of 67 patients with osteoarthritis (OA) were also included in the study. Control PBMCs and sera were obtained from a group of 88 healthy individuals who were matched for sex ratio and mean age with the patient group from the same hospitals and who had not received immunosuppressive or immunomodulatory drugs for various reasons for at least 2 months before the time of sample collection. Informed consent was obtained from all subjects before sample collection. The study protocol and consent form were approved by the Institutional Medical Ethics Review Board of Soochow University. SF was centrifuged at 350 × *g* for 3 minutes, and supernatants were collected and immediately stored at −80 °C until use. Mononuclear cells were prepared by Ficoll-Hypaque separation (GE Healthcare Life Sciences, Little Chalfont, UK) in all cases from blood specimens of patients with RA and controls using the standard protocol.

**Table 1:** Clinical information about patients with RA and controls

Group	RA	OA	HC
Sample size, *n*	83	67	88
Age, yr, mean ± standard deviation	58.30 ± 13.01	56.78 ± 13.98	55.73 ± 15.19
Sex, *n*			
Male	22	17	20
Female	61	50	68
Mean duration of disease, mo (range)	53.27 (1–456)	46.31 (1–323)	–
Stages of disease, *n*			
Early RA (≤12 mo)	30		
Late RA (>12 mo)	53		
Activity of disease, *n*			
Remission (DAS28 < 2.6)	10	–	–
Low (2.6 ≤ DAS28 ≤ 3.2)	16	–	–
Moderate (3.2 < DAS28 ≤ 5.1)	45	–	–
High (DAS28 > 5.1)	12	–	–
Manifestations of disease, *n*			
Extraarticular	23	–	–
Limited joint manifestations	60	–	–
Drug use before study	–	–	–

*RA* rheumatoid arthritis, *OA* osteoarthritis, *HC* healthy controls, *DAS28* 28-joint Disease Activity Score

### Preparation of mononuclear cells and isolation of CD4^+^ T lymphocytes

PBMCs were isolated from patients with RA or controls. Cells were washed by centrifugation in RPMI 1640 medium and subsequently resuspended in cold phosphate-buffered saline (PBS) containing 2.5 % fetal bovine serum (FBS) at a cell density of 1 × 10^7^/ml. CD4^+^ T cells were prepared from freshly isolated PBMCs by depleting cells expressing CD8, CD14, CD16, CD19, CD36, CD56, CD123, γ/δ-T-cell receptors, and glycophorin A using No-Touch T-cell isolation kits (Miltenyi Biotec, Bergisch Gladbach, Germany). The purity of CD4^+^ T cells was 95–98 % as determined by flow cytometry using specific antibodies.

### T-cell stimulation

Purified T-cell preparations derived from peripheral blood of patients with RA or controls were cultured at 1 × 10^6^/ml in RPMI 1640 medium containing 10 % FBS in the presence of 1 μg/ml anti-CD3 monoclonal antibody (mAb) (clone OKT-3; eBioscience, San Diego, CA, USA) and 0.05 μg/ml anti-CD28 mAb (clone CD28.2; eBioscience). Cells were maintained at 37 °C in a 5 % CO_2_ atmosphere for 48 h and were then harvested for RNA extraction before real-time polymerase chain reaction (PCR) analysis.

### Induction of the expression of PD-1 isoforms

PBMCs from healthy individuals were cultured in 24-well plates at 1 × 10^6^/ml in RPMI 1640 medium containing 10 % FBS in the presence or absence of a panel of recombinant human cytokines [interferon (IFN)-γ, interleukin (IL)-17A, or tumor necrosis factor (TNF)-α; R&D Systems, Minneapolis, MN, USA), respectively, at the indicated concentrations. Cells were kept in culture at 37 °C in a 5 % CO_2_ atmosphere for 12 h or 24 h and were then harvested for isolation of CD4^+^ T cells before real-time PCR analysis.

### RNA extraction and real-time PCR

Total RNA was isolated from cell pellets using an RNeasy Mini Kit (Qiagen, Hilden, Germany). Genomic DNA was removed using RNase-Free DNase (Qiagen). RNA was stored at −80 °C. First-strand cDNA synthesis was performed using a Sensiscript Reverse Transcription Kit (Qiagen) with random hexamers as the primers. mRNA expression of the genes encoding IFN-γ, TNF-α, IL-2, IL-10, IL-17A, IL-21, flPD-1, and PD-1Δex3 mRNA was determined by real-time PCR using SYBR Green Master Mix (Applied Biosystems, Foster City, CA, USA). Data were collected and quantitatively analyzed on an ABI PRISM 7900 sequence detection system (Applied Biosystems). The *GAPDH* gene was used as an endogenous control to normalize for differences in the amount of total RNA in each sample.

### Detection of soluble PD-1 and cytokine molecules by ELISA

Concentrations of sPD-1 were measured quantitatively in SF and sera using ELISA according to our established protocol. For sPD-1, Costar ELISA 96-well plates (Fisher Scientific, Pittsburgh, PA, USA) were precoated with the capture anti-PD-1 mAb (4B9) [31, 34] at 3 μg/ml in 0.05 M carbonate buffer solution (pH 9.6) overnight at 4 °C. The coating solution was aspirated off, and unoccupied binding sites on the plates were blocked with 2 % bovine serum albumin in PBS at 37 °C for 1 h. After being washed three times with PBS containing 0.2 % Tween-20, samples and standards (PD-1 fusion proteins; R&D Systems) were added to the wells for 2 h at 37 °C in duplicate. The specific binding protein was detected with biotinylated anti-PD-1 mAb (bio-9H1, 1 μg/ml) [31, 34] for 1 h at 37 °C, followed by streptavidin-horseradish peroxidase at 1:2000 for 1 h at 37 °C, and then revealed with the substrate 3,3′,5,5′-tetramethylbenzidine (Sigma-Aldrich, St. Louis, MO, USA). The reaction was stopped with 2 M H_2_SO_4_, and the plates were analyzed at 450 nm using a microplate reader (Bio-Rad Laboratories, Hercules, CA, USA). The plates were washed five times with PBS containing 0.2 % Tween-20 after each step. The serial twofold dilutions of the soluble CD28-Fc, PD-L1-Fc, and PD-L2-Fc proteins starting from 100 ng/ml were detected by the ELISA to assess the specificity of the established system. For the detection of serum expression of TNF-α, IFN-γ, and IL-17A, high-sensitivity ELISA kits for soluble cytokines were obtained from eBioscience and used according to the manufacturer’s instructions.

### Assessment of PD-1 function

The effects of PD-1 crosslinking during T-cell activation were determined according to the method described by Bertsias et al. [20]. CD4^+^ T cells (1 × 10^5^/well) stimulated in 96-well plates with plate-bound anti-CD3 (1 μg/ml) and soluble anti-CD28 (250 ng/ml) were incubated with a pool of L929-PD-L1 cells (PD-L1 transgenic cell line) or with various concentrations of plate-bound PD-L1-Fc (0.5 μg/ml; R&D Systems), a chimeric protein containing the extracellular part of human PD-L1 linked to the Fc fragment of human immunoglobulin G1 (IgG1). Then the above CD4^+^ T cells were incubated in the presence or absence of the recombinant fusion protein PD-1-Fc for 4 days. After 3 days of coculture supernatants were collected for cytokine measurements, and after 4 days cells were pulsed with the reagent of Cell Counting Kit-8 (CCK-8; Dojindo Molecular Technologies, Kumamoto, Japan) for another 4 h to measure proliferation.

### Induction of CIA and treatment of DBA/1 mice with PD-1-Fc

Male DBA/1 mice were purchased from the Chinese Academy of Sciences (Beijing, China) and maintained in a specific pathogen-free animal facility at Soochow University. All animal procedures were approved by the Institutional Animal Care and Use Committee of Soochow University for the use of laboratory animals. CIA was induced according to the standard protocol. Briefly, an emulsion was formed by dissolving 2 mg/ml chick type II collagen (CII; Sigma-Aldrich) overnight at 4 °C in 10 mM acetic acid and combining it with an equal volume of complete Freund’s adjuvant containing 5 mg/ml heat-killed *Mycobacterium tuberculosis* (Difco H37Ra; BD Diagnostics, Sparks, MD, USA). Eight-week-old mice were injected intradermally at two sites in the base of the tail with a total of 100 μl of emulsion. This was repeated as a booster injection 21 days later. In some experiments, DBA/1 mice received intraperitoneal injections of a high dose of PD-1-Fc (0.15 mg/mouse, *n* = 7) or a low dose of PD-1-Fc (0.05 mg/mouse, *n* = 7) on days 1, 3, and 5 postimmunization. PD-1-Fc protein consists of the extracellular domains of murine PD-1 linked to the Fc fragment of mouse IgG1. Animals were assessed for redness and swelling of all four limbs, and a clinical score ranging from 0 (no inflammation) to 4 (extensive swelling and erythema of the entire paw) was assigned to each mouse two or three times per week for up to 42 days. After the mice were killed, their rear paws were removed, fixed, decalcified, and embedded in paraffin. Frontal sections of the paw tissue (5 mm) were stained with hematoxylin and eosin and evaluated according to the presence or absence of inflammatory cell infiltrates (defined as focal accumulations of leukocytes).

### CII-specific T-cell proliferation and cytokine production

Five days or ten weeks after the second immunization, the spleen was removed. Single-cell suspensions of erythrocyte-depleted splenocytes were prepared in RPMI 1640 medium supplemented with 10 % FBS, 2 mM glutamine, 1 mM sodium pyruvate, and antibiotics. Whole splenocytes were seeded into 96-well flat-bottom microtiter plates and cultured in the presence or absence of the indicated amounts of denatured (60 °C, 30 minutes) bovine CII for 72 h. PD-1-Fc protein was added at the start of the assay. CCK-8 was added (10 μl/well), and incubation was continued for 2 h, followed by measurement of absorbance at 450 nm. Supernatants from similar cultures were collected after 96 h for assessment of cytokine production using a cytometric bead array (CBA; BD Biosciences, San Jose, CA, USA).

### Cytometric bead array

Cytokine concentrations in supernatants were determined using a mouse Th1/Th2/Th17 CBA kit (BD Biosciences), which allowed for the simultaneous detection of IL-2, IL-4, IL-6, IL-10, TNF-α, IFN-γ, and IL-17A. Aliquoted samples were thawed, and CBA analysis was performed according to the manufacturer’s protocol. Briefly, beads coated with capture antibodies were mixed, and 50 μl of the capture bead mixture was added to 50 μl of sample. To these sample bead complexes, 50 μl of phycoerythrin (PE)-conjugated detection antibody was added, and this mixture was incubated for 3 h in the dark at room temperature. Samples were washed with 1 ml of wash buffer at 1100 rpm for 5 minutes, and the pellets were resuspended in 300 ml of wash buffer. Cytokine standards were serially diluted to facilitate the construction of calibration curves necessary for determining protein concentrations of test samples. Flow cytometric analysis was performed on a BD FACSCanto II (BD Immunocytometry Systems, Erembodegem, Belgium) with BD FACSDIVA version 6 software, and data were analyzed with FCS Express version 3 software (De Novo Software, Glendale, CA, USA).

### Antibodies and flow cytometry

Human cells were stained with the following antibodies: fluorescein isothiocyanate (FITC)-anti-CD4, PE-cyanine 7 (Cy7)-conjugated anti-IFN-γ, anti-IL-4, anti-IL-17A, and anti-TNF-α (all from BioLegend, San Diego, CA, USA). Mouse cells were stained with FITC-conjugated anti-CD4, PE-anti-chemokine (C-X-C) motif receptor 5, PE-Cy7-conjugated anti-inducible costimulatory molecule, anti-IFN-γ, anti-IL-4, anti-IL-17A, and anti-TNF-α (all from BioLegend).

Intracellular staining was performed as follows. PBMCs or splenocytes were stimulated with 50 ng/ml phorbol 12-myristate 13-acetate, 750 ng/ml ionomycin (both from Sigma-Aldrich), and 1 μl/ml GolgiStop (BD Biosciences) for 5 h at 37 °C. Surface staining was performed for 20 minutes with FITC anti-human/anti-mouse CD4 antibody on ice. Cells were washed and resuspended in fixation/permeabilization solution (BD Cytofix/Cytoperm kit; BD Biosciences) and stained with PE-Cy7-conjugated anti-IFN-γ, anti-IL-4, anti-IL-17A, and anti-TNF-α (all from BioLegend) for flow cytometric analysis. PE-Cy7-conjugated IgG1 and FITC-conjugated IgG1 (BD Biosciences) were used as isotype controls. All data were analyzed using FlowJo software (FlowJo, Ashland, OR, USA).

### Computed tomographic scanning

Micro–computed tomography was performed using a cone beam scanner (μCT 20; SCANCO Medical, Brüttisellen, Switzerland) with a fixed x-ray fan beam of 7-μm spot size at 50 kVp and 160 mA. Integration time was 140 milliseconds, and slices were scanned at high resolution (1024 × 1024–pixel matrix per slice) and size of 25 microvoxels.

### Histological and radiological assessments of arthritis

CIA mice were killed at day 35 or day 60. Anteroposterior radiographs of the four limbs were obtained with a cabinet soft x-ray apparatus (CMB-2; Softex, Tokyo, Japan). The hind paws were then removed, fixed in formalin, decalcified in 10 % ethylenediaminetetraacetic acid, embedded in paraffin, sectioned, and stained with hematoxylin and eosin.

### Statistical analysis

All statistical analyses were performed using IBM SPSS 20.0 for Windows software (IBM, Armonk, NY, USA). All the quantitative data are presented as mean ± standard deviation. Student’s *t* test was used to analyze differences between the groups. A Mann–Whitney *U* test based on nonparametric analysis was performed for independent samples. A paired-samples *t* test or nonparametric Wilcoxon signed-rank test was performed for paired samples. For multiple comparisons, one-way analysis of variance or the Kruskal–Wallis test was initially performed to determine whether an overall statistically significant change existed before using the paired or unpaired Student’s *t* test. For correlation analyses, a Spearman’s *r* value derived from Pearson’s *r* was calculated. A *p* value less than 0.05 was considered statistically significant.

## Results

### sPD-1 levels correlate with clinical parameters and cytokine concentration in sera and SF of patients with RA

**Fig. 1:**
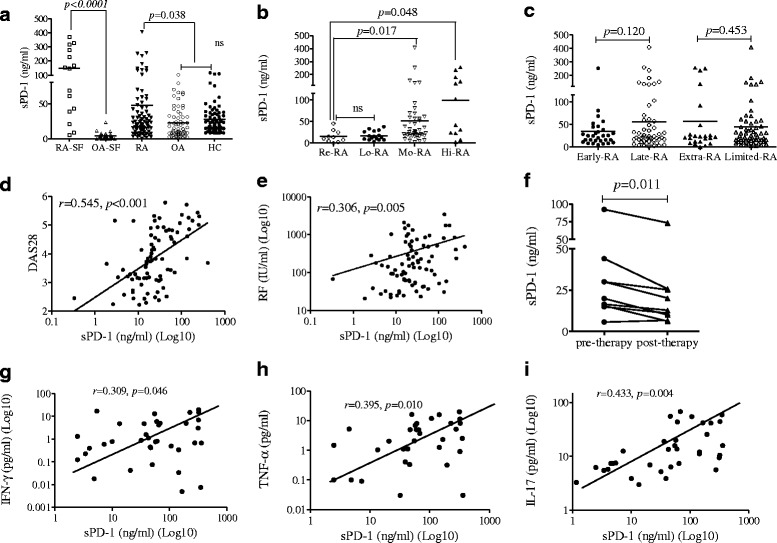
Detection of soluble programmed cell death 1 (sPD-1) protein in synovial fluid (SF) and serum of patients with rheumatoid arthritis (RA) and controls. **a** Concentrations of sPD-1 were analyzed in SF or serum of patients with RA or controls [osteoarthritis (OA) or healthy controls (HC)] by enzyme-linked immunosorbent assay. *Horizontal bars* represent the mean concentration within a given group. **b** The levels of sPD-1 in serum specimens from patients with moderate rheumatoid arthritis activity (Mo-RA; 3.2 < 28-joint Disease Activity Score ≤ 5.1) (*p* = 0.017) and patients with high rheumatoid arthritis activity (Hi-RA; 28-joint Disease Activity Score >5.1) (*p* = 0.048) were higher than those in remission (Re-RA) or those with low rheumatoid arthritis activity (Lo-RA; 2.6 ≤ 28-joint Disease Activity Score ≤ 3.2). **c** The levels of sPD-1 in serum specimens of patients with RA did not differ significantly between those with late RA (Late-RA; >12 months duration) and patients with extraarticular manifestations (Extra-RA) or between those early in the disease course (Early-RA; ≤12 months duration) and patients with limited joint manifestations (Limited-RA). **d** and **e** Correlation of sPD-1 levels with disease activity [28-joint Disease Activity Score (DAS28)] (*r* = 0.545, *p* < 0.001) and rheumatoid factor (RF) content (*r* = 0.306, *p* = 0.005). **f** sPD-1 levels were significantly reduced after methotrexate therapy (*p* = 0.011). **g**–**i** Correlation between serum concentrations of sPD-1 and tumor necrosis factor (TNF)-α (*r* = 0.309, *p* = 0.046), interferon (IFN)-γ (*r* = 0.395, *p* = 0.010), and interleukin (IL)-17A (*r* = 0.433, *p* = 0.006) in patients with RA. The *r* value indicates the calculated regression coefficient

Higher concentrations of sPD-1 were detected in the SF and serum of patients with RA than in OA-SF (*p* < 0.0001) and control serum specimens (*p* = 0.038) (Fig. 1a). The levels of sPD-1 in serum specimens from Mo-RA (*p* = 0.017) and Hi-RA (*p* = 0.048) patients were higher than those in Re-RA patients and Lo-RA patients (Fig. 1b). Clinical analyses showed that sPD-1 levels were correlated with DAS28 (*r* = 0.545, *p* < 0.001) and RF content (*r* = 0.306, *p* = 0.005) (Fig. 1d, e). MTX treatment significantly reduced sPD-1 level compared with pretherapy levels (*p* = 0.011) (Fig. 1f). In addition, sPD-1 levels in RA serum samples correlated significantly with the concentrations of TNF-α, IFN-γ, and IL-17A in the same serum samples (Fig. 1g–i). These results suggest that sPD-1 is aberrantly expressed in RA serum, and the level of sPD-1 correlates with the serum concentrations of TNF-α, IFN-γ, and IL-17A. Taken together, these results suggest that high levels of circulating sPD-1 are observed in RA and may be associated with disease severity and activity.

### Induction of PD-1Δex3 splicing variants by proinflammatory cytokines

**Fig. 2:**
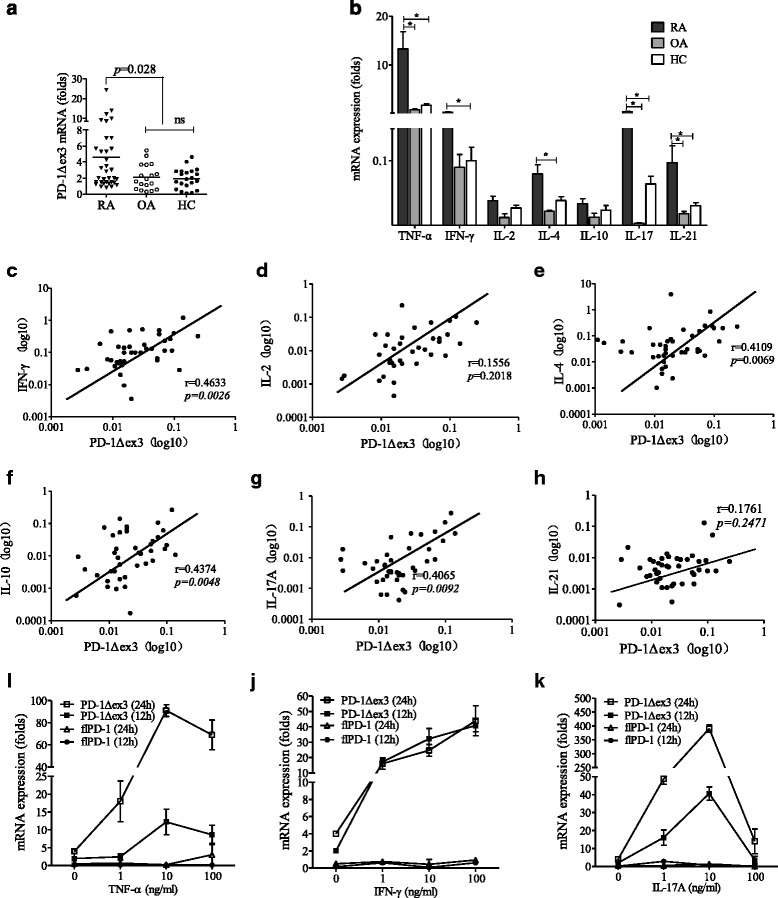
Expression of the programmed cell death 1 protein lacking exon 3 (PD-1Δex3) splicing variant in T cells and correlation between PD-1Δex3 messenger RNA (mRNA) levels and cytokines. **a** Expression of PD-1Δex3 was analyzed in peripheral blood mononuclear cells (PBMCs) of patients with rheumatoid arthritis (RA) (*n* = 34) or controls [patients with osteoarthritis (OA), *n* = 18; healthy controls (HC), *n* = 21) by real-time polymerase chain reaction (RT-PCR). Horizontal bars represent the mean mRNA level within a given group. **b** Expression of cytokines was analyzed in PBMCs of patients with RA or controls (OA or HC) by RT-PCR. *Asterisks* indicate statistically significant differences between the groups (*p* < 0.05). **c**–**h** Correlation of PD-1Δex3 mRNA levels with cytokine mRNA levels. The *r* value indicates the calculated regression coefficient. **i**–**k** Mononuclear cells from healthy volunteers were incubated with 0–100 ng/ml of the indicated recombinant human cytokines for 12 h and 24 h. CD4^+^ T cells were then purified for RT-PCR analysis. *flPD-1* full-length isoform of programmed cell death 1 protein, *TNF-α* tumor necrosis factor α, *IFN-γ* interferon γ, *IL* interleukin

Next, we examined the expression of PD-1Δex3. The PD-1Δex3 splice variant lacks the membrane-spanning domain but has an unchanged extracellular domain, suggesting that the putative translational product is sPD-1 [25]. We found that patients with RA had increased expression of PD-1Δex3 compared with patients with OA and healthy controls (*p* < 0.028) (Fig. 2a). It has been shown that proinflammatory cytokines might be responsible for the progression of arthritis [6]. We found that the mRNA levels of the genes encoding the proinflammatory cytokines IFN-γ, TNF-α, IL-4, IL-21, and IL-17A were higher in the PBMCs of patients with RA (Fig. 2b), and the IFN-γ, TNF-α, IL-10, and IL-17A mRNA levels were positively correlated with the mRNA level of PD-1Δex3 (Fig. 2c–h). We hypothesized that these proinflammatory cytokines, which are expressed abundantly in RA serum, might be responsible for the induction of increased expression of PD-1Δex3 in T cells in RA. Thus, we analyzed the ability of the proinflammatory cytokines IFN-γ, TNF-α, and IL-17A to induce expression of PD-1Δex3 in vitro in PBMCs obtained from healthy individuals. As shown in (Fig. 2i–k), the expression of PD-1Δex3 in T cells could be selectively induced by IFN-γ, TNF-α, and IL-17A in a dose-dependent manner, whereas the expression of flPD-1 was not significantly affected by the addition of the indicated cytokines.

### sPD-1 block PD-1 pathway in vitro

**Fig. 3:**
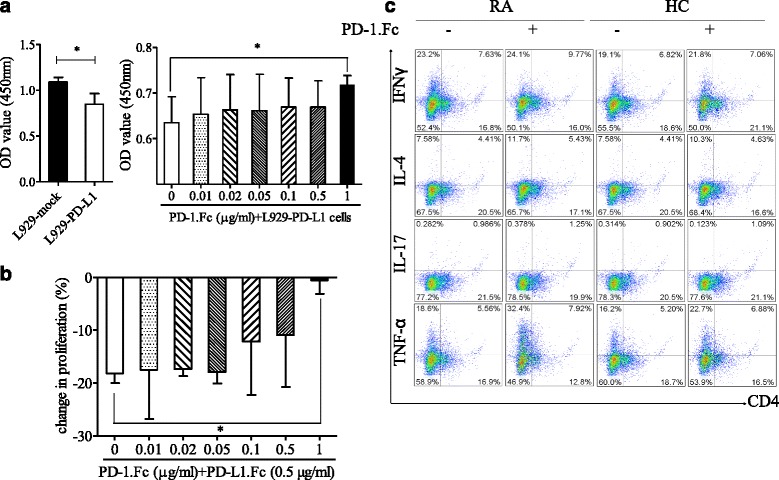
In vitro blocking using PD-1-Fc increases the proliferative and cytokine-producing capacity of CD4^+^ T cells from patients with rheumatoid arthritis (RA). **a** and **b** CD4^+^ T cells were incubated with ligands L929-PD-L1 cells or PD-L1-Fc in the presence or absence of PD-1-Fc for 4 days. The in vitro proliferative response of CD4^+^ T cells from patients with RA to L929-PD-L1 cells or to PD-L1-Fc (0.5 μg/ml) was analyzed in the absence or presence of PD-1-Fc (0–1 μg/ml) using the Cell Counting Kit-8 method. *OD* optical density. The *p* value was calculated using the Mann–Whitney *U* test. Values are the mean ± standard deviation. *p < 0.05 versus controls. **c** Aliquots of peripheral blood mononuclear cells from patients with RA (*n* = 5) and healthy controls (HC) (*n* = 5) were incubated with a pool of CD3 antibody in the presence or absence of PD-1-Fc for 4 days. Brefeldin A was added for the last 16 h. The cells were then collected and stained for the cell surface markers CD4 and PD-1 and for the intracellular cytokines interferon (IFN)-γ, tumor necrosis factor (TNF)-α, interleukin (IL)-4, and IL-17A after fixation and permeabilization. *PD-1* programmed cell death 1

CD4^+^ T cells were incubated with a pool of L929-PD-L1 cells or with PD-L1-Fc protein in the presence or absence of the recombinant fusion protein PD-1-Fc (PD-1-Fc) for 4 days, then cocultured with CCK-8 for the proliferation assay. PD-1-Fc promoted proliferation of CD4^+^ T cells cocultured with PD-L1 transgenic cells (*p* = 0.0074) (Fig. 3a). Meanwhile, T-cell suppression by PD-L1-Fc was neutralized by PD-1-Fc (*p* < 0.05) (Fig. 3b). PBMCs from patients with RA and healthy controls were incubated with anti-CD3 in the presence or absence of PD-1-Fc for 4 days with the addition of brefeldin A for the last 16 h. The cells were then collected and stained for the cell surface marker CD4, as well as for the intracellular cytokines IFN-γ, TNF-α, IL-4, and IL-17 after fixation/permeabilization. We found that PD-1-Fc increased the percentage of CD4^+^IFN-γ^+^ and CD4^+^IL-17^+^ cells (Fig. 3c), suggesting that sPD-1 has the ability to promote Th1/Th17 cells, which are critical pathogenic T cells in autoimmune diseases, including RA.

### Systemic administration of sPD-1 to CII-immunized mice accelerates arthritis onset and joint damage, and increases autoantibody production and Th1/Th17 responses

**Fig. 4:**
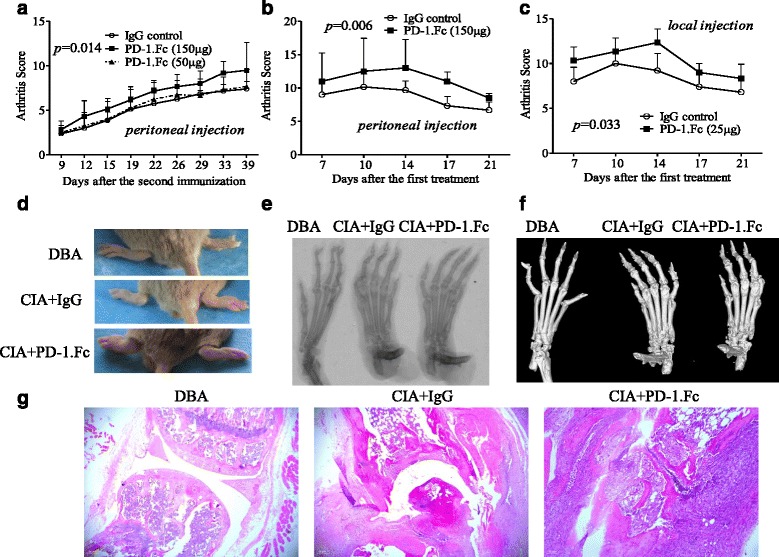
Systemic administration of PD-1-Fc to type II collagen (CII)-immunized mice accelerates arthritis onset and joint damage. **a** Mean clinical scores of arthritis for all four limbs in collagen-induced arthritis (CIA) mice that received 50 μg (*n* = 7) or 150 μg (*n* = 7) of PD-1-Fc in phosphate-buffered saline (PBS) intravenously for 5 consecutive days beginning on day 1 after the second immunization with CII. Adjuvant-immunized controls were treated with immunoglobulin G (IgG) (*n* = 7). **b** Mean clinical scores of arthritis for all four limbs in CIA mice that received 50 μg (*n* = 5) or 150 μg (*n* = 5) of PD-1-Fc in PBS intravenously for 5 consecutive days beginning on day 1 after the first treatment. Controls were treated with IgG (*n* = 5). **c** Mean clinical scores of arthritis for all four limbs in CIA mice that received 25 μg of PD-1-Fc in PBS locally injected into the limbs (*n* = 3) for 3 consecutive days beginning on day 1 after the first treatment. Controls were treated with IgG (*n* = 3). **d** Photographs of CIA mice on day 39 after the second CII immunization. These animals were treated with PD-1-Fc (*lower* and *middle photographs*) or IgG (*top photograph*). **e** and **f** Representative computed tomographic scans of hind paws from the experiment shown in (**a**) at day 39 after the second immunization. **g** Representative hematoxylin and eosin–stained joint sections from the experiment shown in (**a**) on day 50 after the second immunization. *PD-1* programmed cell death 1 protein

To directly assess the role of sPD-1 in inflammatory arthritis in vivo, CIA was induced in DBA/1 mice, followed by intraperitoneal injection with either soluble murine PD-1-Fc fusion protein or PBS as a control. PD-1-Fc-treated mice developed more severe arthritis. Administration of only three doses of PD-1-Fc, starting at day 1 after the second immunization, markedly exacerbated arthritis progression associated with severe synovial hyperplasia, cartilage damage, and bone erosion (Fig. 4a, g). On computed tomographic scans, we detected obvious narrowing of the joint space and bone erosion compared with IgG-treated controls (Fig. 4e, f). To evaluate whether PD-1-Fc could affect established arthritis, we treated mice with established inflamed limbs at a clinical score of 8.0 ± 2.0 with PD-1-Fc. PD-1-Fc rapidly enhanced arthritic progression with significantly increased joint damage (Fig. 4b, c).

**Fig. 5:**
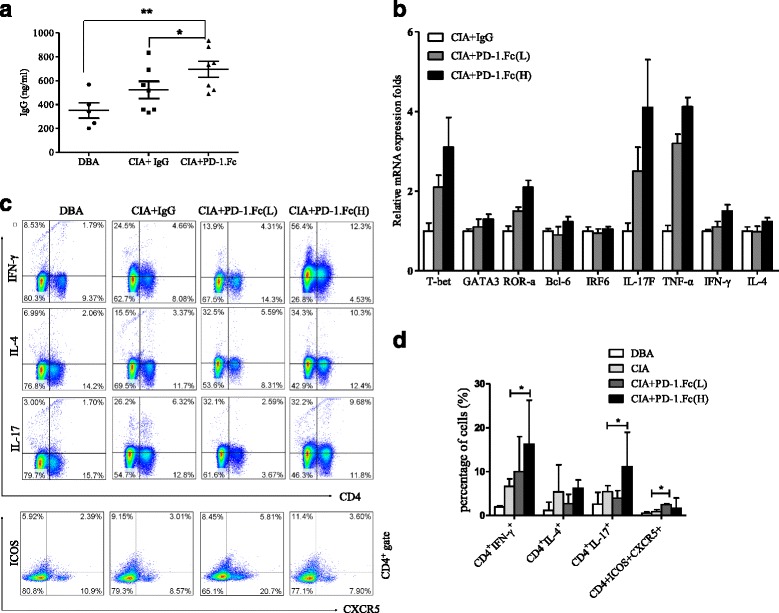
Systemic administration of PD-1-Fc to type II collagen (CII)-immunized mice accelerates arthritis onset, increases autoantibody production, and elevates Th1/Th17 responses. **a** Serum levels of anti-CII autoantibodies were determined by performing enzyme-linked immunosorbent assays in DBA/1 and collagen-induced arthritis (CIA) mice treated with phosphate-buffered saline or PD-1-Fc on day 50 after the second immunization. Values are the mean ± standard deviation (SD). *p < 0.05, **p < 0.01 versus controls. **b** Expression of T-bet, GATA3, RAR-related orphan receptor α (ROR-a), Bcl-6, interferon regulatory transcription factor (IRF)-6, interleukin (IL)-17 F, tumor necrosis factor (TNF)-α, interferon (IFN)-γ, and IL-4 was determined using real-time polymerase chain reactions in splenocytes obtained from each mouse and normalized to β-actin expression. Values are the mean ± SD (*n* = 3 samples per group) and are representative of at least three independent experiments with similar results. **c** and **d** Cells were isolated from the spleen on day 20 of the experiment shown in Fig. 4a and stimulated immediately with phorbol 12-myristate 13-acetate and ionomycin in the presence of GolgiStop before intracellular staining for IL-17A, IFN-γ, and IL-4. Cells were gated on the CD4^+^ T-cell population and analyzed by flow cytometry. Representative dot plots from four sets of similar results are shown. Bars show the mean and SD. *p < 0.05. *PD-1* programmed cell death 1 protein, *CXCR5* chemokine (C-X-C) motif receptor 5, *ICOS* inducible costimulatory molecule

Immunization with CII elicits a specific humoral response in all mice. Although levels of anti-CII antibody are not strictly correlated to arthritis scores, they are usually high in sera from severely diseased mice. Serum levels of anti-CII autoantibody in DBA/1 and CIA mice treated with PBS or PD-1-Fc were analyzed using ELISA at day 50 after the second immunization. The PD-1-Fc-treated mice had significantly increased serum levels of anti-CII autoantibody compared with the controls (*p* < 0.05) (Fig. 5a). Analysis of spleen cells from PD-1-Fc–treated mice revealed significantly upregulated mRNA levels of TNF-α, IFN-γ, and IL-17 F. Expression of RAR-related orphan receptor α and T-bet mRNA was also markedly increased (Fig. 5b). In the PD-1-Fc–treated group, we found significantly increased numbers of Th1 and Th17 cells in the spleen (Fig. 5c, d).

**Fig. 6:**
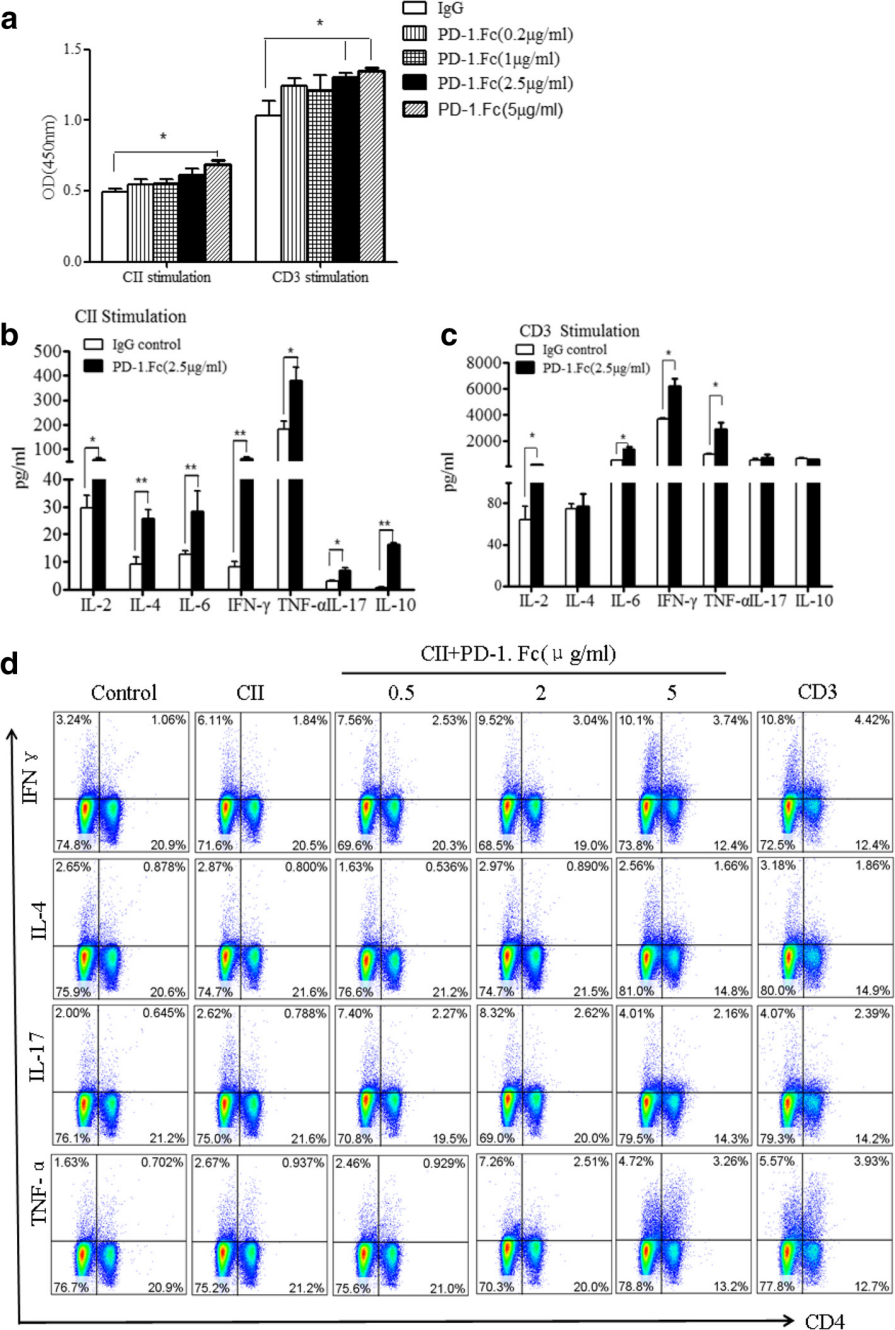
PD-1-Fc promotes the expansion of Th1 and Th17 cells in vitro. **a** The proliferation rates of spleen lymphocytes from collagen-induced arthritis (CIA) mice cultured with type II collagen (CII) or CD3 and PD-1-Fc were determined by Cell Counting Kit-8 assay. Bars show the mean and standard error of the mean. *p < 0.05 versus control. **b** and **c** Cytokine levels in supernatants of spleen lymphocytes from CIA mice that were stimulated with CII or CD3 antibody in the presence of PD-1-Fc, as determined by cytometric bead array. Data are representative of three individual sample analyses. Bars show the mean and standard deviation (SD). *p < 0.05. **d** Percentage of Th1, Th2, and Th17 cells in CD4^+^ T cells from spleen lymphocytes of CIA mice with or without PD-1-Fc induction. Percentages were determined by intracellular staining. Data are representative of three individual sample analyses. Bars show the mean and SD. *p < 0.05 versus control. *IFN* interferon, *IgG* immunoglobulin G, *IL* interleukin, *OD* optical density, *PD-1* programmed cell death 1 protein, *TNF* tumor necrosis factor

To better understand how PD-1-Fc influences the T-cell response, we studied the effect of PD-1-Fc on the proliferation and cytokine secretion of splenocytes in vitro. We cultured splenocytes from CIA mice with or without PD-1-Fc and found that PD-1-Fc treatment enhanced the ability of splenocytes to proliferate (Fig. 6a). After 4 days of culture IL-2, IL-4, IL-6, TNF-α, IL-17A, and IFN-γ, levels were quantified in the supernatants. As shown in Fig. 6b, one of the main effects of PD-1-Fc was to increase the production of IL-2, IL-6, TNF-α, IL-17A, and IFN-γ proteins. IL-4 production was not modified in the presence of PD-1-Fc (Fig. 6b, c). These results were further confirmed by intracellular cytokine staining (Fig. 6d). These results suggest that PD-1-Fc promotes CIA by enhancing Th1 and Th17 responses.

## Discussion

PD-1 and its ligands PD-L1 and PD-L2 are critical for tolerance and immune homeostasis by inhibiting T-cell activation [10, 12]. PD-1^−/−^ mice develop many kinds of autoimmune diseases [14–16].

Recent reports showed that sPD-1 can block the PD-1/PD-L1 pathway in regulating T-cell function during chronic infection, antitumor immunity, and autoimmune diseases [26–29]. He et al. found that sPD-1 can bind PD-1 ligands, block PD-1/ligand interactions, and enhance the cytotoxicity of tumor-specific CTLs [28]. sPD-1 rescues the proliferative response of simian immunodeficiency virus–specific CD4^+^ and CD8^+^ T cells during chronic infection [29]. Previously, we demonstrated the overexpression of PD-1 on CD4^+^ and CD8^+^ T cells and elevated serum levels of sPD-1 in patients with aplastic anemia [31]. We propose that upregulation of sPD-1 molecules might block the PD-1/PD-L1 signaling pathway. Few data are available concerning serum sPD-1 in RA. Recent studies demonstrated that synovial and serum sPD-1 levels are elevated in patients with RA and are correlated with titers of RF [32, 33]. However, the expression and clinical significance of sPD-1 in RA are not well known.

We therefore first explored the clinical significance of sPD-1 in patients with RA by determining the levels of sPD-1 in serum samples. Both sPD-1 levels in serum samples and PD-1Δex3 mRNA expression in PBMCs from patients with RA were elevated compared with those from patients with OA and healthy controls. Clinical analyses showed that sPD-1 levels were closely correlated with disease activity and RF content in patients with RA. The significant correlation between sPD-1 and the DAS28 suggests that sPD-1 may be a marker of disease activity. This result confirms a previous study showing that circulating sPD-1 is derived, at least in part, from inflamed synovium [31] and that serum sPD-1 may reflect RA disease activity because it is associated with synovial inflammation observed on clinical examination [32]. Our findings suggest that aberrant overexpression of sPD-1 might block the PD-1/PD-L1 inhibitory pathway and may be associated with persistent activation of self-reactive T cells in RA, leading to long-term disease progression. We also showed that the PD-1Δex3 splice variant encoding for sPD-1 is specifically associated with RA but is not related to the T-cell activation state, because expression of this variant is markedly increased in T cells derived from RA [31].

A significant portion of this study was devoted to investigating the molecular mechanism of higher expression of sPD-1 in activated CD4^+^ T cells. It was previously shown that proinflammatory cytokines might be responsible for the progression of arthritis. We found that the proinflammatory cytokines produced abundantly in RA serum might be responsible for inducing the increased expression of PD-1Δex3 in CD4^+^ T cells. Upregulated expression of PD-1 in RA does not appear to suppress the function of T cells. We therefore hypothesized that the expected function of PD-1 overexpression in T cells was neutralized. To test this hypothesis, we examined the effect of a recombinant fusion protein containing the extracellular domain of PD-1 (as encoded by the PD-1Δex3 variant) on T-cell proliferation and in the CIA mouse model. Addition of PD-1 fusion protein enhanced T-cell proliferation in vitro (*p* < 0.05). This finding indicated that the regulatory properties of membrane-bound PD-1 and PD-L1 were altered by the presence of their soluble forms in the experimental system.

PD-1 blockade was found to shift antigen-induced cellular reactivity toward a proinflammatory Th1/Th17 response, as evidenced by enhanced production of IFN-γ, IL-2, TNF-α, IL-6, and IL-17A, and to reduce production of the Th2 cytokines IL-5 and IL-13 [35]. We showed that PD-1-Fc enhanced the severity of autoimmune arthritis in mice with CIA. Additionally, levels of pathogenic IgG autoantibody to mouse type II collagen were increased in PD-1-Fc-treated CIA mice, and levels of Th1 and Th17 cells were increased in the spleen, suggesting that the T cells may have systemic effects on the B-cell response as well as local effects on the inflammatory environment. This work demonstrates that CD4^+^ T cells specific for sPD-1 can amplify disease severity after onset of CIA.

## Conclusions

We provide evidence that sPD-1 plays a key inhibitory role in the PD-1/PD-L1 pathway in regulating T-cell function in RA. sPD-1 occurred at high concentrations in the serum and SF of patients with RA. It blocked the regulatory effect of membrane-bound PD-1 on T-cell activation. We identified IFN-γ, TNF-α, and IL-17A as inducers of sPD-1. sPD-1 aggravated progression of CIA through induction of Th1/Th17 responses. sPD-1 regulated peripheral T-cell responses in both human and murine RA. Thus, sPD-1 may represent an additional target for immunomodulatory therapy in RA.

## Abbreviations

CBA: cytometric bead array
CIA: collagen-induced arthritis
CII: type II collagen
CXCR5: chemokine (C-X-C) motif receptor 5
Cy7: cyanine 7
DAS28: 28-joint Disease Activity Score
ELISA: enzyme-linked immunosorbent assay
FBS: fetal bovine serum
FITC: fluorescein isothiocyanate
HC: healthy control
Hi-RA: high rheumatoid arthritis activity (28-joint Disease Activity Score >5.1)
ICOS: inducible costimulatory molecule
IFN: interferon
Ig: immunoglobulin
IL: interleukin
IRF: interferon regulatory transcription factor
Lo-RA: low rheumatoid arthritis activity (2.6 ≤ 28-joint Disease Activity Score ≤ 3.2)
mAb: monoclonal antibody
Mo-RA: moderate rheumatoid arthritis activity (3.2 < 28-joint Disease Activity Score ≤ 5.1)
mRNA: messenger RNA
MTX: methotrexate
OA: osteoarthritis
OD: optical density
PBMC: peripheral blood mononuclear cell
PBS: phosphate-buffered saline
PCR: polymerase chain reaction
PD-1: programmed cell death 1
PE: phycoerythrin
qRT-PCR: quantitative real-time reverse transcription polymerase chain reaction
RA: rheumatoid arthritis
Re-RA: rheumatoid arthritis in remission (28-joint Disease Activity Score <2.6)
RF: rheumatoid factor
ROR-α: RAR-related orphan receptor α, SD, standard deviation
SF: synovial fluid
TNF: tumor necrosis factor
